# Celastrol Supplementation Ablates Sexual Dimorphism of Abdominal Aortic Aneurysm Formation in Mice

**DOI:** 10.3390/biom13040603

**Published:** 2023-03-27

**Authors:** Aida Javidan, Weihua Jiang, Lihua Yang, Ana Clara Frony, Venkateswaran Subramanian

**Affiliations:** 1 Saha Cardiovascular Research Center, University of Kentucky, Lexington, KY 40536, USA; 2 Department of Medicine, Division of Cardiovascular Medicine, University of Missouri, Columbia, MO 65212, USA; 3 Department of Physiology, University of Kentucky, Lexington, KY 40536, USA

**Keywords:** angiotensin II, Celastrol, aneurysm, elastin

## Abstract

Background: Abdominal aortic aneurysms (AAAs) are permanent dilations of the abdominal aorta with 4–5 times greater prevalence in males than in females. The aim of this study is to define whether Celastrol, a pentacyclic triterpene from the root extracts of *Tripterygium wilfordii*, supplementation influences angiotensin II (AngII)-induced AAAs in hypercholesterolemic mice. Methods: Age-matched (8–12 weeks old) male and female low-density lipoprotein (Ldl) receptor-deficient mice were fed a fat-enriched diet supplemented with or without Celastrol (10 mg/kg/day) for five weeks. After one week of diet feeding, mice were infused with either saline (*n* = 5 per group) or AngII (500 or 1000 ng/kg/min, *n* = 12–15 per group) for 28 days. Results: Dietary supplementation of Celastrol profoundly increased AngII-induced abdominal aortic luminal dilation and external aortic width in male mice as measured by ultrasonography and ex vivo measurement, with a significant increase in incidence compared to the control group. Celastrol supplementation in female mice resulted in significantly increased AngII-induced AAA formation and incidence. In addition, Celastrol supplementation significantly increased AngII-induced aortic medial elastin degradation accompanied by significant aortic MMP9 activation in both male and female mice compared to saline and AngII controls. Conclusions: Celastrol supplementation to Ldl receptor-deficient mice ablates sexual dimorphism and promotes AngII-induced AAA formation, which is associated with increased MMP9 activation and aortic medial destruction.

## 1. Introduction

Abdominal aortic aneurysms (AAAs), a permanent dilation of the abdominal aorta, have no medical therapies and have a greater than 80% mortality after rupture [1]. AAA is a sexually dimorphic disease, and AAA incidence in humans and mice is 4–5 fold greater in males than females [2]. AAA is positively associated with aging and smoking, with relatively weak genetic associations. AAA formation involves a complex process of destruction of aortic media and supporting lamina through the activation of matrix metalloproteinases (MMPs) and degradation of extracellular matrix proteins such as elastin [3]. Chronic infusion of angiotensin-II (AngII), a major bioactive peptide of the renin–angiotensin system (RAS), into hypercholesterolemic mice for 28 days via osmotic minipump leads to the development of AAAs only in male mice in a dose-dependent manner [4,5]. Infusion of AngII at the dose of either 500 or 1000 ng/kg/min for 28 days showed an AAA incidence of 30–40% or 60–80%, respectively, in male hypercholesterolemic mice, whereas it showed 0–10% of AAA incidence in female mice [4,5]. However, AngII infusion for 28 days promotes atherosclerosis in both male and female hypercholesterolemic mice [4,5]. AngII-induced atherosclerosis is characterized by an intimal macrophage infiltration that becomes engorged with lipids [6]. AngII-induced AAAs recapitulate major features of human AAAs, mainly male-gender specificity [7]. Understanding the underlying mechanisms and identifying pharmacological agents that regulate AngII-induced AAA may prove to be clinically invaluable to identify a potential target/agent that can halt or suppress AAA progression.

Celastrol, a pentacyclic triterpene from the root extracts of Thunder God Vine (*Tripterygium wilfordii*), is widely used in chronic inflammatory and immunity disorders [8,9,10]. Recently, Celastrol supplementation was shown to reduce AngII-induced cardiac remodeling in mice [11]. The purpose of this study aimed to investigate the effect of Celastrol supplementation on Ang II-induced AAA formation in male and female hypercholesterolemic mice.

## 2. Materials and Methods

### 2.1. Mice and Study Protocol

Age-matched (8–12 weeks old) male (*n* = 5–13) and female (*n* = 5–15) Ldl receptor-deficient mice (colony bred from original stock from The Jackson Laboratory; stock # 002207; backcrossed to a C57Bl/6N mice; B6NTac; Taconic Biosciences, Cambridge City, IN, USA) were used in the present study. All study procedures were approved by the University of Kentucky Institutional Animal Care and Use Committee (protocol # 2011-0907 and 2020-3634). This study followed the recommendations of The Guide for the Care and Use of Laboratory Animals (National Institutes of Health). Ldl receptor genotyping was performed as described previously [12]. The mice were randomly placed by Division of Laboratory Animal Research (DLAR) staff in cages (*n* = 5/cage) upon weaning. The inclusion and exclusion criteria were followed as below:

Inclusion criteria: body weight > 15 g and 8 weeks of age.

Exclusion criteria: body weight < 15 g and <8 weeks of age. Medical cases not related to aortic pathologies were reported by a DLAR veterinarian.

### 2.2. Diet and AngII Infusions

Male and female Ldl receptor -/- mice were fed a saturated fat-enriched diet (21% *wt*/*wt* milk fat; TD.170793, Envigo, Madison, WI, USA) supplemented with or without Celastrol (10 mg/kg/day, BOC Sciences, Shirley, NY, USA) for five weeks. The Celastrol was mixed with high-fat diet in the ratio of 80 ppm, which contains 80 mg of Celastrol/kg. The average weight of high-fat diet pellets was 1 g, and the average diet consumption per mouse was 2–3 pellets (i.e.,) 2–3 g/day. Based on the dose of 10 mg/kg/day, the average dose of Celastrol for a single mouse of 25–30 g body weight was 0.25–0.3 mg/day. The concentration of Celastrol in a 2–3 g high-fat diet was 0.24–0.3 mg, which is equivalent to the dose of 10 mg/kg/day. After one week of diet feeding, mice were infused with either saline or AngII (500 or 1000 ng/kg/min, Bachem, Torrance, CA, USA) for 28 days by Alzet osmotic minipumps (Model 1004 and 2004, Durect Corporation, Cupertino, CA, USA) as described previously [12].

### 2.3. Blood Pressure Measurement

Systolic blood pressure (SBP) was measured on conscious mice using a computerized tail-cuff blood pressure system (Kent Scientific Corp, Torrington, CT, USA) [13]. SBP was measured three consecutive days before pump implantation and during the final week of AngII infusion.

### 2.4. Measurement of Plasma Cholesterol

Total plasma cholesterol concentrations were measured using the enzymatic assay kit (Pointe Scientific Kit) as described previously [12].

### 2.5. Quantification of AAAs by Ultrasound and Ex Vivo AAA Measurements

Luminal dilation of the suprarenal abdominal aorta was measured on days 0, 14, and 28 of saline or AngII infusion by a high-frequency ultrasound imaging system (Vevo 2100, Visual Sonics, Toronto, ON, Canada) using an MS550 MicroScanTM transducer with a resolution of 18–38 MHz. Mice were anesthetized and restrained in a supine position to acquire ultrasonic images. Short axis scans of abdominal aortas were performed from the left renal arterial branch to the suprarenal region. External abdominal aortic width was measured at the endpoint. After saline was perfused through the left ventricle of the heart, the aortas were removed from the origin to iliac bifurcation and placed in formalin (10% *wt*/*vol*) overnight. Adventitial fat was cleaned from the aortas. Aortic images were taken using a Nikon dissecting microscope. AAAs were quantified ex vivo by measuring the maximum external width of the suprarenal abdominal aortic diameter using computerized morphometry (Image-Pro Cybernetics, Bethesda, MD, USA) as described previously [14]. Aneurysm incidence was quantified based on a definition of an external width of the suprarenal aorta that was increased by 50% or greater compared with aortas from saline-infused mice [15]. In addition, AAA rupture is also included in the AAA incidence calculation. For ascending AA measurement, intimal areas of ascending aortas were measured from the ascending aorta to the subclavian branch using Image-Pro Plus software [14,16].

### 2.6. Quantification of Atherosclerosis

Atherosclerosis was quantified on aortic arches as lesion area and percent lesion area on the intimal surface by en face analysis as described previously [5]. Lesion areas were measured using Image-Pro Plus software (MediaCybernetics, Bethesda, MD, USA) by direct visualization of lesions under a dissecting microscope.

### 2.7. Histology and Immunohistochemistry

Abdominal sections of the aorta were fixed, embedded in paraffin, and serially sectioned (5 µm thickness/section) in sets of 10 slides with 2 sections/slide using a microtome. Tissue sections were deparaffinized and stained with Verhoeff’s Van Gieson stain to visualize elastin breaks and Picrosirius Red stain (PSR-2, ScyTek Laboratories, West Logan, UT, USA) to visualize collagen content. Elastin fragmentation was defined as the presence of discernable breaks of the elastic lamina and counted in the aorta sections stained with Verhoeff’s Van Gieson.

Immunohistochemical staining was performed to detect aortic medial smooth muscle cell actin using rabbit anti-mouse antibodies against alpha-smooth muscle cell actin (Abcam, catalog No: ab5694). Biotinylated secondary antibodies from the appropriate species were used (Vector Laboratories, Newark, CA, USA). A peroxidase-based ABC system (Vector Laboratories) and the red chromogen AEC were used to detect the antigen–antibody reaction. Immunostaining was performed with appropriate negative controls, as described previously [17]. The alpha-smooth muscle cell actin positive area of the aortic media or Picrosirius Red stain positive collagen area of the adventitia was quantified in stained aortic sections using Nikon element analysis software as described previously [18]. Briefly, stained sections were imaged using a 20× objective lens from edge to edge. Using the NIS-Elements analysis software, the defined region of interest (ROI) is subtracted with the white color. Under the defined threshold, the ratio of binary area (µm^2^)/ROI area (µm^2^) was calculated.

### 2.8. Gelatin Zymography and Western Blot Analyses

Male and female mice (*n* = 3–5 mice/group) were infused with saline or AngII (500 and 1000 ng/kg/day) for 14 days. Aortic protein (10 µg) was extracted from the abdominal aorta, and matrix metalloproteinases (MMP) activity was examined by in-gel zymography as described previously [19]. Abdominal aortic protein extracts were resolved by using SDS-PAGE (7.5%) in the presence of gelatin (2 mg/mL) to measure the activities of MMP2 and MMP9. Gels were washed with 2.5% Triton X-100 (1 h) followed by distilled water (30 min) and incubated overnight (37 °C) in Tris buffer containing 5 mM calcium chloride. Gels were stained with Coomassie Brilliant Blue for 30 min followed by destaining with acetic acid (7% *vol*/*vol*) and methanol (40% *vol*/*vol*). Gel images were captured using a Bio-Rad Imager, and unstained, translucent, digested regions represent areas of MMP activity. The MMP activity bands were quantified using the Bio-Rad analysis software. An equal amount of protein loading was verified by resolving the aortic lysates (10 µg) by SDS-PAGE (6% *wt*/*vol*) and transferred electrophoretically to PVDF membranes. After blocking with non-dry fat milk (5% *wt*/*vol*), the membranes were probed with antibodies against β-actin (catalog No: A5441, Sigma-Aldrich, St. Louis, MO, USA). Membranes were then incubated with appropriate secondary antibodies, and immune complexes were visualized by chemiluminescence (Pierce, Rockford, IL, USA) and quantified using Bio-Rad analysis software. The MMPs activity was normalized to the loading control, β-actin.

### 2.9. Statistical Analyses

Data are represented as mean ± SEM. Statistical analyses were performed using SigmaPlot 14.5 (Systat Software Inc., Sanjose, CA, USA). Data were tested for normality and equal variance using the Shapiro–Wilk and Brown–Forsythe tests. For two-group comparisons, the unpaired Student’s *t* test was performed for normally distributed and equally variant values, while the Mann–Whitney rank sum test was performed for variables not passing the normality or equal variance test. One- or two-way ANOVA with Holm–Sidak post hoc analyses were performed for multiple-group and multiple-manipulation analysis. Fisher’s exact probability test was used to determine differences between groups in the incidence of aneurysm formation and mortality due to rupture. Repeated measurement data were analyzed with SAS fitting a linear mixed model expressing the temporal trend in systolic blood pressure as a quadratic polynomial in time for each treatment. Values of *p* < 0.05 were considered statistically significant.

## 3. Results

### 3.1. Celastrol Supplementation Increased AngII-Induced AAA in Male Mice

To evaluate the effect of Celastrol supplementation on AngII-induced AAA formation in hypercholesterolemic mice, we initiated a pilot study utilizing male Ldl receptor -/- mice (8 weeks old; *n* = 12–13 per group). In this study, mice were fed a saturated fat-enriched diet supplemented with or without Celastrol (10 mg/kg/day) for five weeks. After one week of the diet, mice were infused with a high dose of AngII (1000 ng/kg/min) via an osmotic mini-pump and sacrificed on day 28 for AAA assessments. Celastrol supplementation significantly decreased high-fat diet-induced body weight gain and AngII-induced blood pressure compared to the control group (Table 1). Despite the reduced body weight, Celastrol-supplemented mice were active, which is similar to the control group. However, Celastrol supplementation did not affect total plasma cholesterol concentrations (Table 1).

**Figure 1:**
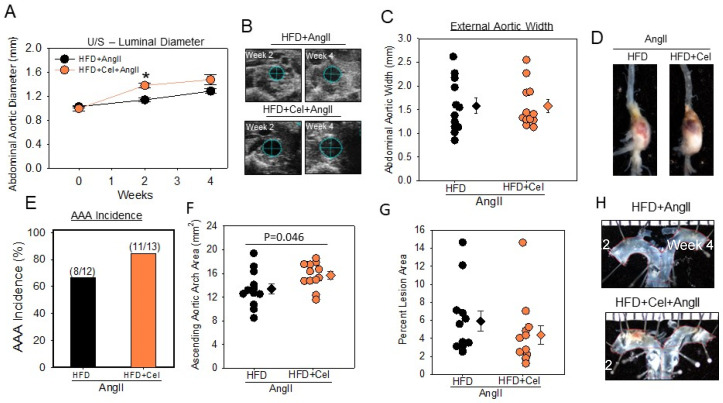
(**A**) Abdominal aortic luminal diameters were measured by ultrasound on weeks 0, 2, and 4 of AngII (1000 ng/kg/min) infusion in male mice (AngII + HFD *n* = 12; AngII + HFD + Cel *n* = 13). (**B**) Ultrasound pictographs of suprarenal aortas of individual mice at weeks 2 and 4 nearest to the mean of the group. (**C**) Measurements of maximal external width of abdominal aortas (AngII + HFD *n* = 12; AngII + HFD + Cel *n* = 13). Black (AngII + HFD) and orange (AngII + HFD + Cel) circles represent individual mice, diamonds represent means, and bars are SEMs. * Denotes *p* < 0.001 when comparing AngII + HFD vs. AngII + HFD + Cel (Student’s *t* test). (**D**) Representative images of abdominal aortas nearest the mean of each group. (**E**) The incidence of AAA in AngII-infused HFD (black bar) or HFD + Cel (orange bar) supplemented groups of mice. Statistical analyses were performed by Fisher’s exact test. (**F**) Area of ascending aortic arch and atherosclerotic lesion area (**G**) was measured on the intimal surface of the aortic arch. *p* = 0.046 when comparing AngII + HFD vs. AngII + HFD + Cel (Student’s *t* test). (**H**) Representative images of ascending aortas nearest the mean of each group. AngII: angiotensin II, HFD: high-fat diet, Cel: Celastrol.

After two weeks of AngII infusion, ultrasonography measurements showed an acceleration in AAA formation in the group receiving Celastrol (Figure 1A,B). Despite the significant difference (*p* < 0.05) between the two groups in week 2, intraluminal measurement by sonography (Figure 1A,B) and maximal aortic width measurements (Figure 1C,D) in week 4 did not show any significant difference between the control and Celastrol groups (Figure 1A–D). AAA incidence rate was similar in both groups (Figure 1E). In addition, Celastrol supplementation showed a modest increase in AngII-induced ascending aortic expansion (Figure 1F,H) but did not affect atherosclerotic lesion areas in aortic arches (Figure 1G,H).

**Figure 2:**
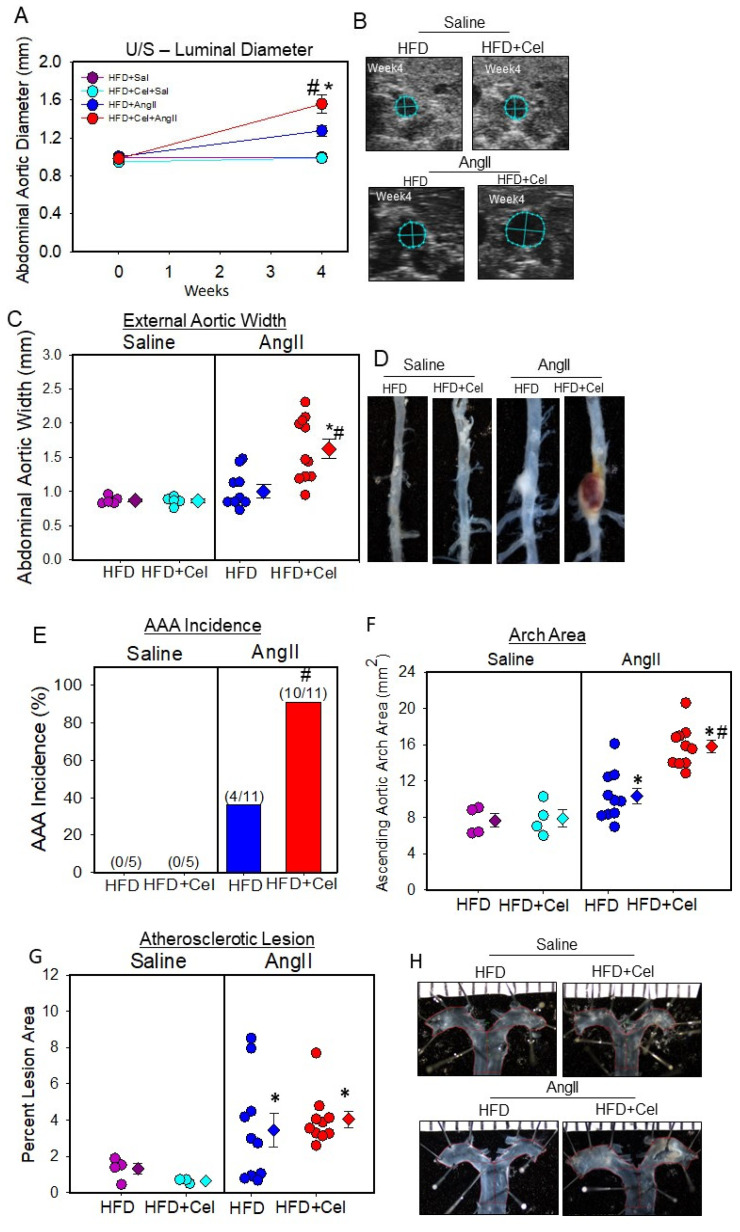
(**A**) Abdominal aortic luminal diameters were measured by ultrasound on weeks 0 and 4 of saline or AngII (500 ng/kg/min) infusion in male mice (saline *n* = 5; AngII *n* = 11). Purple (saline + HFD), teal (saline + HFD + Cel), blue (AngII + HFD), and red (AngII + HFD + Cel) circles represent means, and bars are SEMs. * Denotes *p* < 0.05 when comparing saline vs. AngII infusion; # *p* < 0.05 AngII + HFD vs. AngII + HFD + Cel (two-way ANOVA repeated measures test). (**B**) Ultrasound pictographs of suprarenal aortas of individual mice at week 4 nearest to the mean of the group. (**C**) Measurements of maximal external width of abdominal aortas. * Denotes *p* < 0.001 saline vs. AngII infusion; # *p* < 0.001 AngII + HFD vs. AngII + HFD + Cel (two-way ANOVA with Holm–Sidak post hoc analysis). (**D**) Representative images of abdominal aortas nearest the mean of each group. (**E**) The incidence of AAA. # *p* < 0.001 AngII + HFD vs. AngII + HFD + Cel (Fisher’s exact test). (**F**) Area of ascending aortic arch and atherosclerotic lesion area (**G**) was measured on the intimal surface of the aortic arch. * Denotes *p* < 0.05 when comparing saline vs. AngII infusion; # *p* < 0.05 AngII + HFD vs. AngII + HFD + Cel (two-way ANOVA with Holm–Sidak post hoc analysis). (**H**) Representative images of ascending aortas nearest the mean of each group. AngII: angiotensin II, HFD: high-fat diet, Cel: Celastrol.

Based on the observed early acceleration of AngII-induced aortic dilation at week 2 with Celastrol supplementation and given that AngII promotes AAA severity and incidence in a dose-dependent manner, in the next step, we tested the effect of Celastrol supplementation with a low dose of AngII (500 ng/kg/min) to examine if there is any effect of Celastrol supplementation on the acceleration of a low dose of AngII-induced AAA formation in hypercholesterolemic mice. Male Ldl receptor -/- mice (8–10 weeks old; *n* = 5–13 per group) were fed a saturated fat-enriched diet supplemented with or without Celastrol (10 mg/kg/day) for five weeks. After one week of the diet, mice were infused with either saline or a low dose of AngII (500 ng/kg/min) using osmotic mini-pumps for 28 days. Celastrol supplementation significantly decreased high-fat diet-induced body weight gain in both saline and AngII-infused groups of mice but did not affect total plasma cholesterol concentrations and AngII-induced blood pressure (Table 2). Celastrol-supplemented mice are as active as the control groups of mice. Dietary supplementation of Celastrol significantly promoted a low dose of AngII-induced abdominal aortic luminal dilation (Figure 2A,B) and external aortic width (Figure 2C,D) in male mice as measured by ultrasonography and ex vivo measurement, with 90% AAA incidence (10/11) compared to 36% (4/11) in the control group (Figure 2E). In addition, Celastrol supplementation significantly increased AngII-induced ascending aortic expansion (Figure 2F,H) but did not affect atherosclerotic lesion areas in aortic arches (Figure 2G,H).

### 3.2. Celastrol Supplementation Increased AngII-Induced AAA in Female Mice

Based on the results obtained from male studies, we were interested to see if Celastrol supplementation influenced AAA formation in female Ldl receptor -/- mice. Since female mice are highly resistant to AngII-induced AAA formation, in this study, female Ldl receptor -/- mice were infused with either saline or a high dose of AngII (1000 ng/kg/min) for 4 weeks and fed HFD with or without Celastrol for 5 weeks starting one week before AngII infusion. Celastrol supplementation significantly decreased high-fat diet-induced body weight gain in both saline and AngII-infused groups of mice but did not affect total plasma cholesterol concentrations and AngII-induced blood pressure (Table 3).

**Figure 3:**
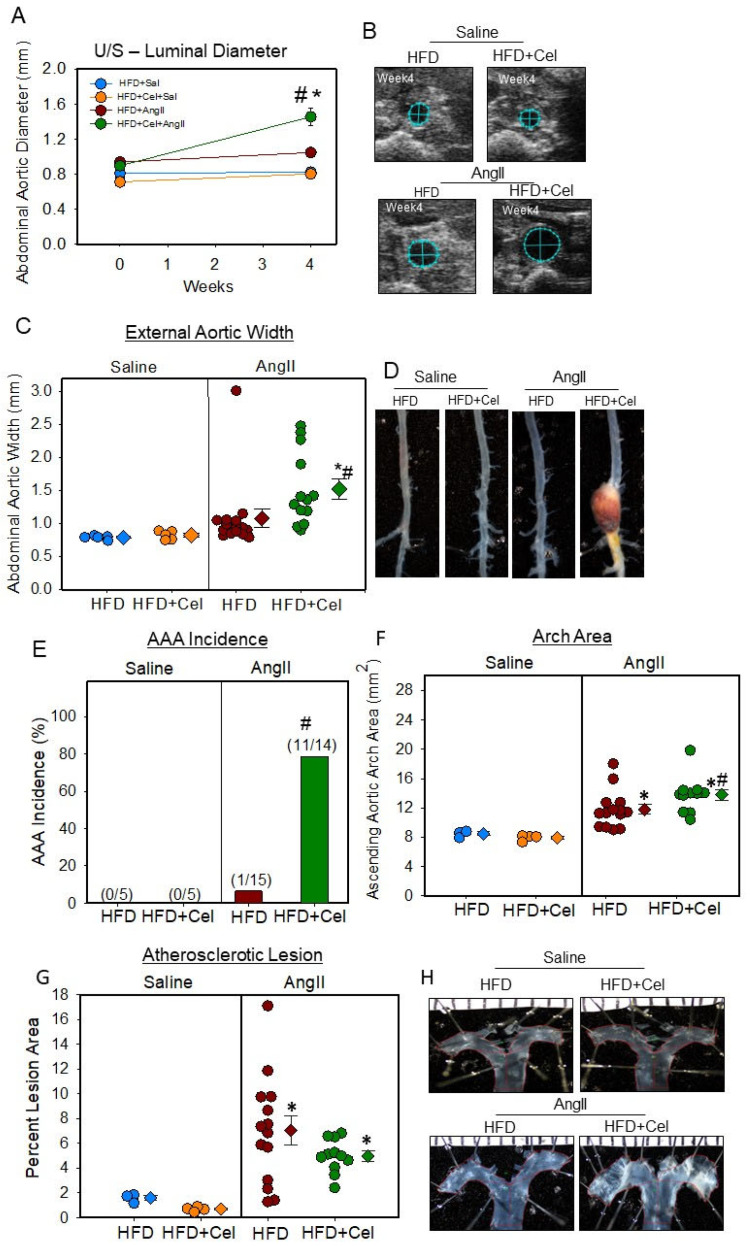
(**A**) Abdominal aortic luminal diameters were measured by ultrasound on weeks 0 and 4 of saline or AngII (1000 ng/kg/min) infusion in female mice (saline *n* = 5; AngII *n* = 13–15). Blue (saline + HFD), orange (saline + Cel), brown (AngII + HFD), and green (AngII + HFD + Cel) circles represent means, and bars are SEMs. * Denotes *p* < 0.05 when comparing saline vs. AngII infusion; # *p* < 0.05 AngII + HFD vs. AngII + HFD + Cel (two-way ANOVA repeated measures test). (**B**) Ultrasound pictographs of suprarenal aortas of individual mice at week 4 nearest to the mean of the group. (**C**) Measurements of maximal external width of abdominal aortas. * Denotes *p* < 0.001 when comparing saline vs. AngII infusion; # *p* < 0.001 AngII + HFD vs. AngII + HFD + Cel (two-way ANOVA with Holm–Sidak post hoc analysis). (**D**) Representative images of abdominal aortas nearest the mean of each group. (**E**) The incidence of AAA in saline or AngII-infused HFD or HFD + Cel-supplemented groups of mice. # *p* < 0.001 AngII + HFD vs. AngII + HFD + Cel (Fisher’s exact test). (**F**) Area of ascending aortic arch and atherosclerotic lesion area (**G**) was measured on the intimal surface of the aortic arch of saline or AngII (1000 ng/kg/min) infused female mice supplemented with high fat diet +/- Celastrol. * Denotes *p* < 0.05 when comparing saline vs. AngII infusion; # *p* < 0.05 AngII + HFD vs. AngII + HFD + Cel (two-way ANOVA with Holm–Sidak post hoc analysis). (**H**) Representative images of ascending aortas nearest the mean of each group. AngII: angiotensin II, HFD: high-fat diet, Cel: Celastrol.

Celastrol supplementation in female mice resulted in a significant increase in AngII-induced abdominal aortic luminal dilation (Figure 3A,B) and external aortic width (Figure 3C,D) as measured by ultrasonography and ex vivo measurement with 80% incidence (11/14) compared to 6% (1/15) in the control group (Figure 3E). In addition, Celastrol supplementation modestly but significantly increased AngII-induced ascending aortic expansion (Figure 3F,H) but did not affect atherosclerotic lesion areas in aortic arches (Figure 3G,H).

### 3.3. Celastrol Supplementation Increased Abdominal Aortic Medial Elastin Break and MMP9 Activity in Both Male and Female Mice Infused with AngII

**Figure 4:**
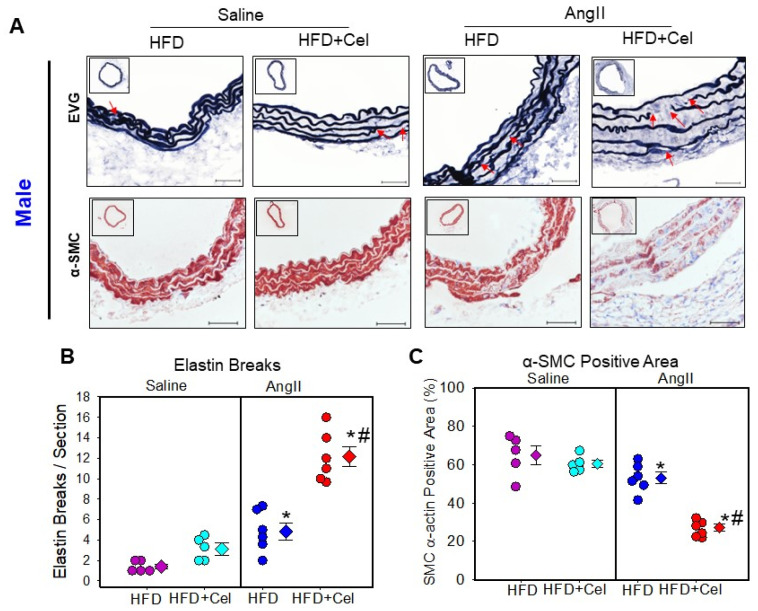
(**A**) Representative suprarenal aortic tissue sections from saline or Ang II (500 ng/kg/min) infused male mice supplemented with high fat diet +/- Celastrol stained with Verhoeff’s or α-SMC actin. Red arrows indicate medial breaks. Scale bars correspond to 50 μm. Measurements of aortic medial break (**B**) and α-SMC actin positive area (**C**) in the cross-sections of abdominal aortas (*n* = 5–6 in each group). Purple (saline + HFD), teal (saline + Cel), blue (AngII + HFD), and red (AngII + HFD + Cel) circles represent individual mice, diamonds represent means, and bars are SEMs. * Denotes *p* < 0.05 when comparing saline vs. AngII infusion; # *p* < 0.05 AngII + HFD vs. AngII + HFD + Cel (two-way ANOVA with Holm–Sidak post hoc analysis). AngII: angiotensin II, HFD: high-fat diet, Cel: Celastrol.

**Figure 5:**
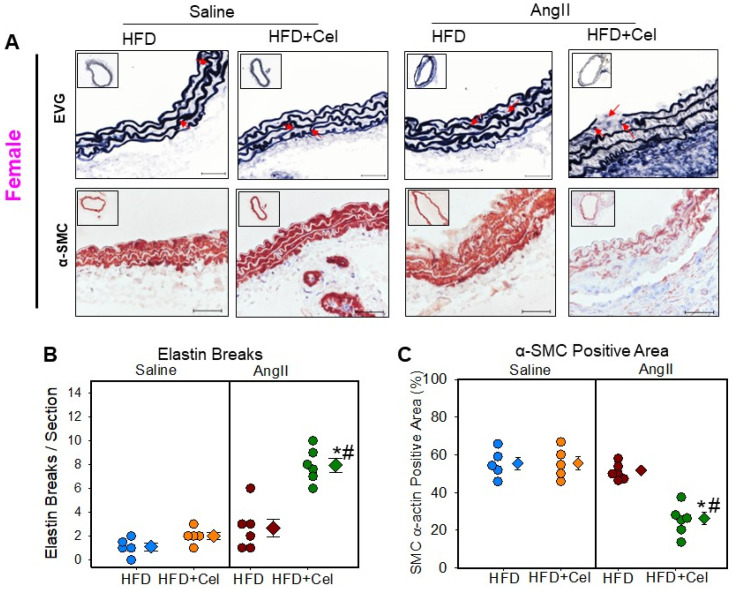
(**A**) Representative suprarenal aortic tissue sections from saline or Ang II (1000 ng/kg/min) infused female mice supplemented with high fat diet +/- Celastrol stained with Verhoeff’s or α-SMC actin. Red arrows indicate medial breaks. Scale bars correspond to 50 μm. Measurements of aortic medial break (**B**) and α-SMC actin positive area (**C**) in the cross-sections of abdominal aortas (*n* = 5–6 in each group). Blue (saline + HFD), orange (saline + Cel), brown (AngII + HFD), and green (AngII + HFD + Cel) circles represent individual mice, diamonds represent means, and bars are SEMs. * Denotes *p* < 0.05 when comparing saline vs. AngII infusion; # *p* < 0.05 AngII + HFD vs. AngII + HFD + Cel (two-way ANOVA with Holm–Sidak post hoc analysis). AngII: angiotensin II, HFD: high-fat diet, Cel: Celastrol.

**Figure 6:**
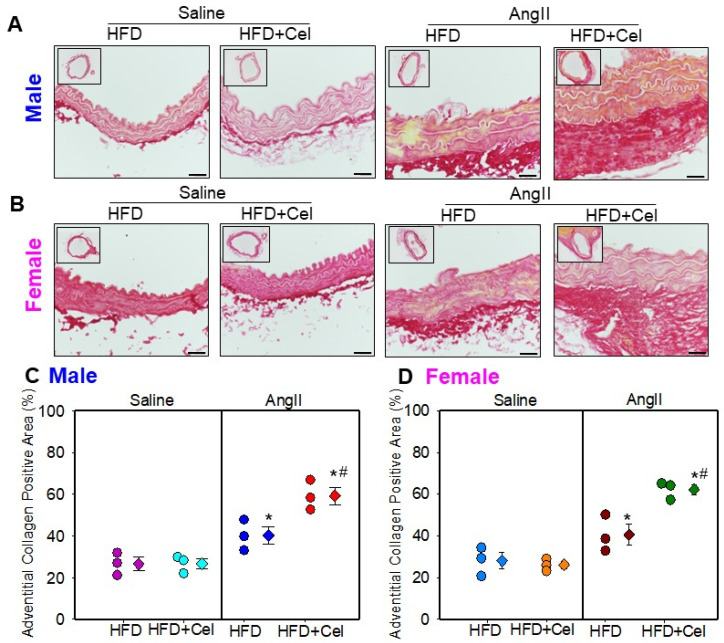
Representative suprarenal aortic tissue sections from saline or Ang II (500 or 1000 ng/kg/min) infused male (**A**) and female (**B**) mice supplemented with high fat diet +/- Celastrol stained with Picrosirius red stain. Scale bars correspond to 50 μm. Measurements of adventitial collagen positive area (Male—(**C**); Female—(**D**)) in the cross-sections of abdominal aortas (*n* = 3 in each group). Male—purple (saline + HFD), teal (saline + Cel), blue (AngII + HFD), and red (AngII + HFD + Cel) and Female—blue (saline + HFD), orange (saline + Cel), brown (AngII + HFD), and green (AngII + HFD+ Cel) circles represent individual mice, diamonds represent means, and bars are SEMs. * Denotes *p* < 0.05 when comparing saline vs. AngII infusion; # *p* < 0.05 AngII + HFD vs. AngII + HFD + Cel (two-way ANOVA with Holm–Sidak post hoc analysis). AngII: angiotensin II, HFD: high-fat diet, Cel: Celastrol.

Histological characterization of abdominal aortic sections obtained from male and female mice showed aortic wall disruption in AngII-infused aortas that received Celastrol supplementation. Verhoeff’s Van Gieson elastin staining and α-smooth muscle cell actin immunohistochemical staining revealed that Celastrol supplementation significantly promoted AngII-induced medial elastin breakage and a significantly reduced medial α-smooth muscle cell actin-positive area in both male (Figure 4A–C) and female (Figure 5A–C) mice. Additionally, Picrosirius red staining analyses revealed that Celastrol supplementation exhibited a significant and pronounced collagen deposition thickening of the adventitia in both male (Figure 6A,C) and female mice (Figure 6B,D).

**Figure 7:**
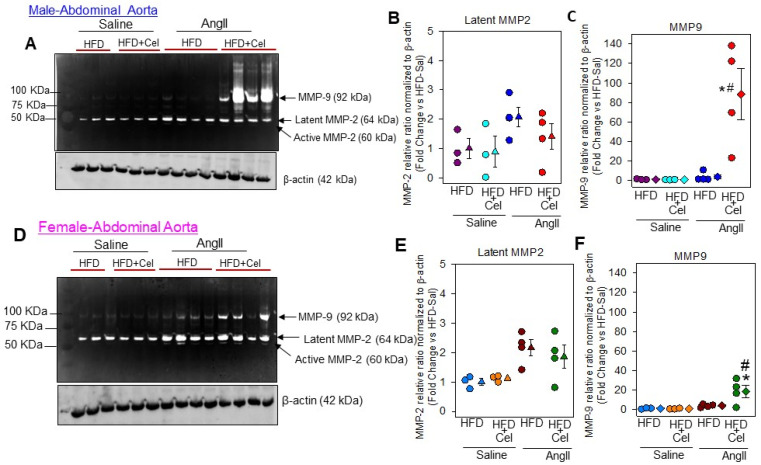
Gelatin zymography detected MMP-2, -9 in abdominal aortas from saline and AngII-infused male (**A**) and female (**D**) mice supplemented with a high-fat diet +/- Celastrol. Measurements of the relative ratio of MMPs in male (**B**,**C**) and female (**E**,**F**) normalized to loading control β-actin protein (*n* = 4). Circles represent individual mice, diamonds represent means, and bars are SEMs. * Denotes *p* < 0.05 when comparing saline vs. AngII infusion; # *p* < 0.05 AngII + HFD vs. AngII + HFD + Cel (two-way ANOVA with Holm–Sidak post hoc analysis). AngII: angiotensin II, HFD: high-fat diet, Cel: Celastrol.

In addition, in-gel gelatin zymographic analyses of abdominal aortic lysates revealed that Celastrol supplementation significantly increased AngII-induced matrix metalloproteinases (MMP)-9, not -2 activities in both male and female mice, compared to AngII and saline controls (Figure 7).

## 4. Discussion

Recent evidence has demonstrated the beneficial effects of Celastrol on transverse aortic constriction (TAC)-induced myocardial fibrosis and attenuating AngII-induced cardiac dysfunction in mice [11,20]. For the first time, our study investigated the effect of Celastrol supplementation on AngII-induced AAA formation in hyperlipidemic mice. The significant finding of this study was that Celastrol supplementation caused the early acceleration of aortic luminal expansion in a high dose of AngII-infused male mice after two weeks but not at the four weeks endpoint. By lowering the dose of AngII in male mice, we demonstrated that Celastrol significantly increased luminal aortic dilation and AAA formation with a higher incidence of AAA than in control groups after 28 days of AngII infusion. Like humans, AAA formation in mice is 4–5 times greater in males than females. By infusion of a high dose of AngII into female mice, only 20–30% of female mice develop aneurysms [21]. Based on this evidence, we were interested in how Celastrol supplementation affects AAA formation in female mice. Our data indicated that Celastrol supplementation combined with AngII infusion resulted in propounding increases in internal luminal dilation and AAA formation in female mice as male mice.

Consistent with the published literature, [22,23] in our study, Celastrol supplementation significantly suppressed Western diet-induced body weight gain. Irrespective of decreased HFD-induced body weight gain, all Celastrol supplemented mice are as active as the control group. The decreased body weight gain observed with Celastrol supplementation could be due to its effect on demonstrated leptin sensitization [22]. However, we did not observe any difference in diet consumption. Celastrol is shown to mediate its anti-obesity effect through the interleukin-1 receptor (IL1R), as IL1R mice are resistant to Celastrol mediated anti-obesity effect [22]. In addition, diet-mediated calorie reduction in mice is shown to reduce AngII-induced AAA formation in ApoE-/- mice through the activation of anti-aging Sirtuin 1 (SIRT1) in vascular SMCs [24]. Celastrol supplementation is shown to ameliorate HFD-induced hepatic steatosis in mice by increasing hepatic SIRT1 [25]. However, in our study, irrespective of body weight loss, Celastrol supplementation significantly increased AngII-induced AAA formation in both male and female Ldlr-/- mice, which suggests that Celastrol mediated AAA acceleration is independent of body weight reduction and possibly independent of SIRT1 induction. To support our hypothesis, in our current study, we observed a significant and dramatic increase in AngII-induced MMP9 activity in Celastrol supplemented AngII-infused hypercholesterolemic male and female mice.

With respect to SBP, by using tail-cuff blood pressure measuring method, we observed a slightly higher SBP in the range of pre-hypertensive in both male and female hypercholesterolemic mice [26]. Although our major objective is to examine the effect of Celastrol on AngII-induced AAAs, the SBP measurement was utilized as a readout of an effective AngII infusion in our experimental study mice [5]. AngII infusion at the dose of 500 ng/kg/min for 28 days significantly increased SBP in male mice compared to baseline. Similarly, a high dose of AngII (1000 ng/kg/min) infusion moderately but significantly increased SBP in female mice. Celastrol supplementation had no further influence on AngII-induced SBP in male and female mice. In addition, Celastrol supplementation had no effect on HFD-induced hypercholesterolemia in both male and female mice.

One of the hallmarks of AAAs is elastin degradation, which leads to aortic dilation and rupture. In hyperlipidemic male mice, the AngII-induced AAA is preceded by aortic dissection, which is characterized by hematoma and thrombi presence in the adventitial side of the abdominal aorta [6]. Although it is not yet clear, it could be the ability of AngII to promote ECM destruction by activating MMPs. Matrix metalloproteases such as MMP2 and MMP9 play a crucial role in AAA formation by degradation of elastin fibers in the aortic media layer [27]. To the best of our knowledge, there is no report available in the literature showing that AngII infusion had any significant effect on aortic medial elastin breaks, MMP9 activity and collagen deposition in female mice. In our study, Celastrol supplementation resulted in significantly increased elastin degradation, which is associated with accelerated MMP9 activation and adventitial collagen deposition in both male and female mice abdominal aortas infused with AngII. Although MMP2 and MMP9 activities increased with AngII infusion compared to saline groups, only MMP9 was significantly upregulated by Celastrol supplementation in both male and female mice. These data strongly suggest that Celastrol supplementation abolished sexual dimorphic effects of AngII by influencing medial elastin breaks, aortic MMP9 activity and adventitial collagen deposition in addition to AAA formation.

The Celastrol supplementation-mediated increase in AngII-induced aortic MMP9 activity may partially explain the mechanism through which Celastrol promotes AngII-induced elastin break and aortic dilation irrespective of decreased HFD-induced body weight gain in both male and female mice. However, in the literature, in several studies focused on different disease conditions such as intrahepatic cholestatic of pregnancy [28], or breast cancer [29,30], Celastrol is shown to suppress MMP-2 and -9 activities, which is contrary to our current observation in AngII-induced AAA model. Our current observations indicate that Celastrol supplementation-mediated AngII increased aortic MMP9 and plays a critical role in alleviating sexual dimorphism of AngII-induced AAA formation in hypercholesterolemic mice. However, the mechanisms by which Celastrol activates MMP9, promotes AAA development, and abolishes sexual dimorphism remains unclear. Therefore, future studies are warranted to focus on cellular and molecular pathways dysregulated by Celastrol treatment in accelerating AngII-induced AAA in both male and female mice.

In the present study, since we used two different doses of AngII (i.e.,), low dose of AngII (500 ng/kg/min) in male mice and a high dose of AngII (1000 ng/kg/min) in highly resistant female mice supplemented with HFD + Celastrol, we cannot directly compare the outcomes of male and female mice. However, our current observation clearly suggests that Celastrol supplementation accelerated AngII-induced AAA formation in highly resistant female hypercholesterolemic mice and abolished the sexual dimorphic effect of AngII-induced AAA in mice.

## 5. Conclusions

In summary, we demonstrated that Celastrol supplementation to hypercholesterolemic mice ablates sexual dimorphism and profoundly increases AngII-induced AAA formation, which is associated with increased MMP activation and aortic medial destruction.

## Figures and Tables

**Table 1:** Effects of Celastrol in male Ldl receptor -/- mice infused with high dose of AngII (1000 ng/kg/min).

Groups	HFD	HFD + Celastrol
Infusion	AngII	AngII
*N*	12	13
Body Weight Pre-Infusion (g)	22.1 ± 0.7	21.9 ± 0.9
Body Weight Post-Infusion (g)	25.1 ± 0.7	18.1 ± 0.6 *
Plasma Cholesterol (mg/dL)	951.5 ± 32.2	961 ± 56.5
Systolic BP Pre-Infusion (mmHg)	138 ± 4	133 ± 4
Systolic BP Post-Infusion (mmHg)	188 ± 5 ^	169 ± 8 ^*

Values are represented as means ± SEMs. Body weights and plasma cholesterol concentrations were determined at termination. Systolic blood pressure was measured prior to (week 0) and during AngII infusion (week 4). * Denotes *p* < 0.05 HFD + Celastrol vs. HFD by Student’s *t* test. ^ Denotes *p* < 0.05 systolic BP post-infusion vs. pre-infusion, by Student’s *t* test.

**Table 2:** Effects of Celastrol in male Ldl receptor -/- mice infused with saline or low dose of AngII (500 ng/kg/min).

Groups	HFD		HFD + Celastrol	
	Saline	AngII	Saline	AngII
*N*	5	10	5	11
Body Weight Pre (g)	27.1 ± 1.1	25.2 ± 1.0	25.3 ± 0.7	26.1 ± 0.3
Body Weight Post (g)	30.8 ± 1.4	29.6 ± 0.8	20.1 ± 1.2 *^$^	20.8 ± 0.4 *^$^
TC (mg/dL)	1192 ± 81	1208 ± 98	1351 ± 89	1437 ± 48
SBP Pre (mmHg)	136 ± 4	146 ± 3	145 ± 4	150 ± 3
SBP Post (mmHg)	154 ± 7	182 ± 6 ^#^	138 ± 4	181 ± 4 ^#^

Values are represented as means ± SEMs. Plasma total cholesterol (TC) concentrations was determined at termination. Body weights and systolic blood pressure (SBP) were measured prior to (week 0) and during AngII infusion (week 4). * Denotes *p* < 0.05 HFD + Celastrol vs. HFD by two-way ANOVA. ^$^ Denotes *p* < 0.05 HFD + Celastrol Pre vs. Post. ^#^ Denotes *p* < 0.05 systolic BP post-infusion vs. pre-infusion, by two-way repeated measures ANOVA.

**Table 3:** Effects of Celastrol in female Ldl receptor -/- mice infused with saline or high dose of AngII (1000 ng/kg/min).

Groups	HFD		HFD + Celastrol	
	Saline	AngII	Saline	AngII
*N*	5	15	5	13
Body Weight Pre (g)	18.9 ± 0.7	19.6 ± 0.3	18.7 ± 0.6	19.2 ± 0.3
Body Weight Post (g)	21.9 ± 0.6	22.8 ± 0.5	16.6 ± 0.9 *^$^	16.7 ± 0.3 *^$^
TC (mg/dL)	982 ± 87	1079 ± 86	1032 ± 87	1147 ± 54
SBP Pre (mmHg)	150 ± 4	139 ± 3	147 ± 3	149 ± 3
SBP Post (mmHg)	169 ± 5 ^#^	166 ± 5 ^#^	133 ± 3	176 ± 4 ^#^

Values are represented as means ± SEMs. Body weights and plasma total cholesterol (TC) concentrations were determined at termination. Systolic blood pressure (SBP) was measured prior to (week 0) and during AngII infusion (week 4). * Denotes *p* < 0.05 Celastrol vs. HFD by two-way ANOVA. ^$^ Denotes *p* < 0.05 HFD + Celastrol Pre vs. Post. ^#^ Denotes *p* < 0.05 systolic BP post-infusion vs. pre-infusion, by two-way repeated measures ANOVA.

## Data Availability

The data sets generated during and/or analyzed during the current study are not publicly available as they have not been anonymized; however, they are available from the corresponding author upon reasonable request.
